# New polybranchiaspiform fishes (Agnatha: Galeaspida) from the Middle Palaeozoic of China and their ecomorphological implications

**DOI:** 10.1371/journal.pone.0202217

**Published:** 2018-09-19

**Authors:** Zhikun Gai, Liwu Lu, Wenjin Zhao, Min Zhu

**Affiliations:** 1 Key Laboratory of Vertebrate Evolution and Human Origins of Chinese Academy of Sciences, Institute of Vertebrate Paleontology and Paleoanthropology, Chinese Academy of Sciences, Beijing, China; 2 CAS Center for Excellence in Life and Paleoenvironment, Beijing, China; 3 University of Chinese Academy of Sciences, Beijing, China; 4 The Geological Museum of China, Beijing, China; SOUTHWEST UNIVERSITY, CHINA

## Abstract

The 438–370-million-year-old galeaspids, diversified armoured jawless vertebrates (‘ostracoderms’) from China and northern Vietnam, were assumed to have a benthic feeding habit in a coastal, marine environment. Here, we describe two new genera of galeaspid fishes, *Platylomaspis* gen. nov. and *Nanningaspis* gen. nov. from the Middle Palaeozoic of China. The two new forms are characterized by a rostral process and strikingly broad ventral rim, and clustered with *Gumuaspis* to form a new family, Gumuaspidae, which represents the most primitive clade of Polybranchiaspiformes. They extend the earliest occurrence of Polybranchiaspiformes backward about 19 million years, and expand its geographical distribution from southern China and northern Vietnam to the Tarim Basin, northwestern China. The new taxa exhibit many morphological convergences with modern rays, and might specify a new kind of lifestyle of galeaspids, the half burrowing habit. Probably benefiting from the new lifestyle, the Gumuaspidae has become the longest lasting galeaspid family. The new findings demonstrated that the demersal galeaspids had developed three different kinds of lifestyles: semi-infaunal benthic (half buried), epibenthic, and suprabenthic (nektonic) habits to accommodate to differentiated ecological niches, and reached the peak of their diversity by the Pragian of the Early Devonian.

## Introduction

The 438–370-million-year-old galeaspids are a diversified but endemic group of armoured jawless vertebrates (‘ostracoderms’) occurring only in China and northern Vietnam [1–5]. Although galeaspids have no biting jaws, they are regarded as an important stem-group of gnathostomes that provides the crucial fossil evidence for the culmination of stepwise anatomical changes towards crown gnathostomes [6–8]. For example, galeaspids have a diagnostic large median dorsal opening for the common nostril of paired separated nasal sacs, which reflects an intermediate condition for the developmental establishment of complete diplorhiny and jaws in jawed vertebrates [1, 2]. For a long time, galeaspids were simply assumed to be benthic jawless fishes in a coastal, marine environment because they bear a strongly flattened head-shield, dorsally-set eyes and nostril, and a ventral mouth [3]. However, a streamlined galeaspid *Rhegmaspis*, which was recently known from the Lower Devonian of Yunnan, displays adaptations for a suprabenthic or nektonic lifestyle probably with more active feeding habit among galeaspids [9]. Here, we further describe two new genera of polybranchiaspiform galeaspids, *Platylomaspis* gen. nov. from the Silurian of the Tarim Basin, Xinjiang, and *Nanningaspis* gen. nov. from the Devonian of Nanning, Guangxi. The two new polybranchiaspiforms exhibit a rostral process and strikingly broad ventral rim, probably denoting a new lifestyle of galeaspids.

## Materials and methods

### Materials

The material of *Platylomaspis serratus* gen. et sp. nov. was collected by Professor Shiben Zhang in 1990 and 1992 when he worked in Tarim Petroleum Exploration and Development Bureau, Korla, Xinjiang. The holotype (GMC V 2415.1) and paratype (GMC V 2415.2) of *Platylomaspis serratus* are permanently housed and accessible for examination in the collections of the Geological Museum of China (GMC), NO.15 Xisi Yangrou Lane, Xicheng District, Beijing, 100034, China. The material of *Nanningaspis zengi* gen. et sp. nov. was collected by Guangchun Zeng in 2017. The holotype of *Nanningaspis zengi* (IVPP V24897) is permanently housed and accessible for examination in the collections of the Institute of Vertebrate Paleontology and Paleoanthropology (IVPP), Chinese Academy of Sciences, NO.142 Xi-zhi-men-wai Street, Xicheng District, Beijing 100044, China. The materials of *Gumuaspis* and *Pseudolaxaspis* gen. nov. in IVPP were examined as well.

**Institutional abbreviations**—IVPP, Institute of Vertebrate Paleontology and Paleoanthropology, Chinese Academy of Sciences; GMC, Geological Museum of China.

### Geological settings

**Fig 1:**
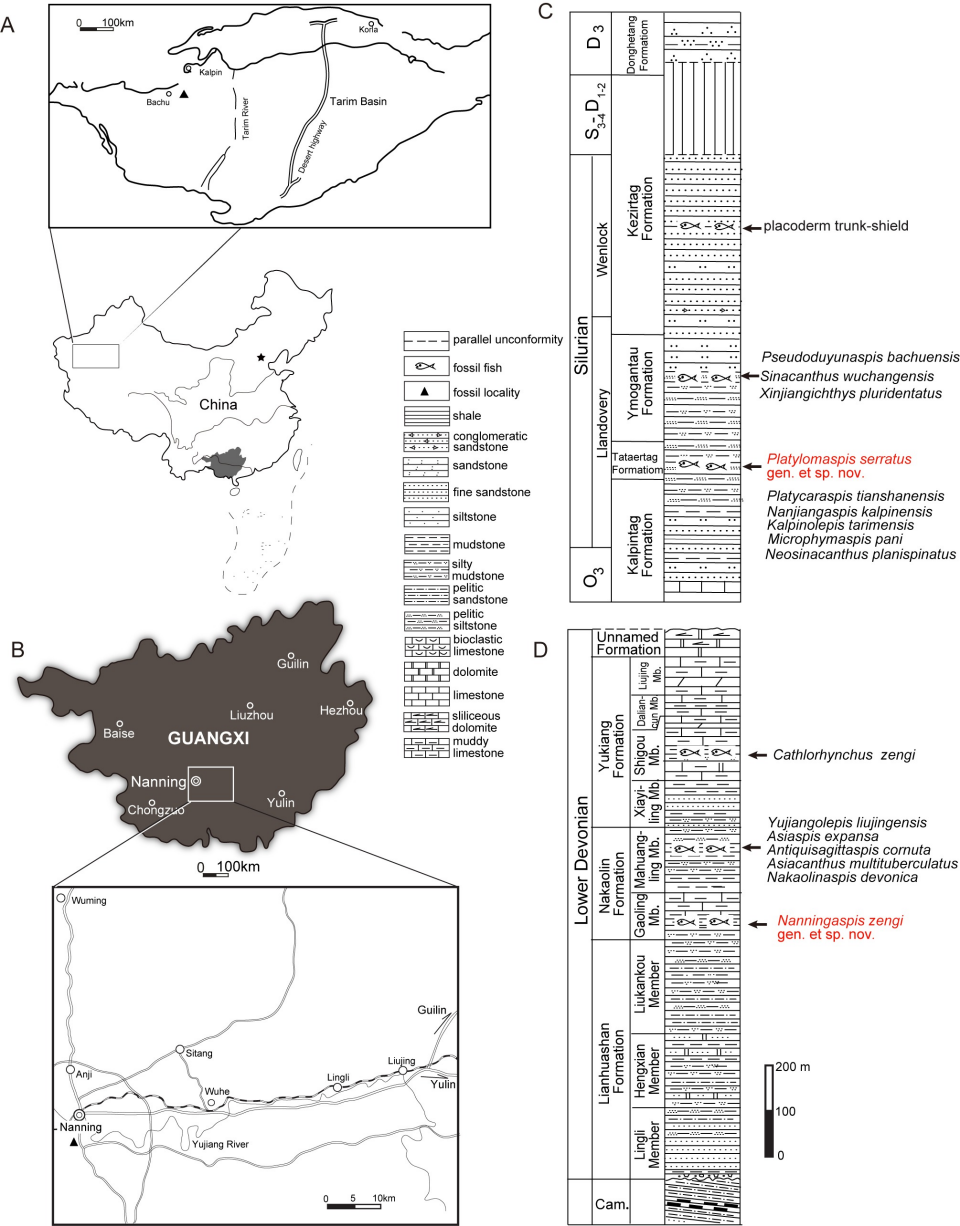
Fish localities and lithological columns of the fish-bearing stratigraphy. A. fossil site of *Platylomaspis serratus* gen. et sp. nov. in the Tarim Basin, Xinjiang; B. fossil site of *Nanningaspis zengi* gen. et sp. nov. in central Guangxi (A, B illustrated by Aijuan Shi); C. lithological columns of the Silurian fish-bearing stratigraphy, modified after Zhao et al.[18]; D. lithological columns of Lower Devonian fish-bearing stratigraphy, modified after Qiao and Zhu[22].

*Platylomaspis* gen. nov. was collected from the Tataertag Formation (Telychian, Llandovery, Silurian) near the Tielikewatie village of Kalpin County, Xinjiang (Fig 1A). The Silurian strata in the north-western margin of the Tarim Basin are subdivided into four formations in ascending order: the Kalpintag, Tataertag, Ymogantau and Kezirtag formations (Fig 1C). In the Kalpin region, the Tataertag Formation is underlain conformably by the Kalpintag Formation and overlain conformably by the Ymogantau Formation. The fish-bearing Tataertag Formation is composed of grey, greyish-white siltstone, sandstone and mudstone intercalated with light-purple, purplish-red siltstone and marlstone, reflecting a shallow-water littoral facies [10]. The Tataertag Formation abounds in the fossilized remains of galeaspids including *Platycaraspis tianshanensis*, *Nanjiangaspis kalpinensis*, *Kalpinolepis tarimensis*, *K*. *zhangi*, *Microphymaspis pani*, *Hanyangaspis* sp., and sinacanths (Chondrichthyes), *Neosinacanthus planispinatus*, *N*. sp., and *Sinacanthus wuchangensis* [11–17]. The fossil fish remains are referred to the *Platycaraspis*—*Kalpinolepis—Sinacanthus* assemblage [18], or the Tataaiertage assemblage [19, 20] which corresponds to the Wentang Assemblage from the Rongxi Formation in South China. In addition to vertebrate remains, the Tataertag Formation is rich in invertebrate brachiopods, gastropods, bivalves, and microfossils (acritarchs, scolecodonts, cryptospores and plant-derived cuticles), most of which were known from the Telychian, Llandovery, Silurian [10]. Hence, the fish-bearing Tataertag Formation could be assigned to the early Telychian (Fig 1C).

*Nanningaspis* gen. nov. was collected from the bottom of the Nakaolin (Nagaoling) Formation (Pragian, Early Devonian), Nanning, Guangxi Zhuang Autonomous Region, South China (Fig 1B). The Early Devonian strata in central region of Guangxi are subdivided into five formations in ascending order: the Lianhuashan, Nakaolin, Yukiang, Moding and Najiao formations (Fig 1D) [21–24]. The Nakaolin Formation is dominated by fine sandstone, siltstone and mudstone, and the fish remains occur in the grayish-green quartziferous siltstones [20]. The formation yielded the vertebrate macroremains including galeaspids *Asiaspis expansa*, *Antiquisagittaspis cornuta*, and arthrodires *Szelepis* sp., *Yujiangolepis liujingensis*, and *Buchanosteus guangxiensis* [25–32]. Some microvertebrate remains are also found in the formation, such as acanthodians *Gomphonchus liujingensis* and *Machaeracanthus*? *bohemicus*, and the sarcopterygian *Onychodus* sp. [30, 33]. The vertebrate assemblage in the Nakaolin Formation was referred to the *Sanchaspis*-*Asiaspis* Assemblage [30] or the *Xujiachong* Assemblage [20] which is also known in the Posongchong Formation (Wenshan, Yunnan), the Xujiachong Formation (Qujing, Yunnan), and the Pingyipu Group (Jiangyou, Sichuan) [20]. The vertebrates are associated with corals, chitinozoans, brachiopods, conodonts [34], plus spores and acritarchs [35], indicating the neritic facies of deposition [30]. Based on the conodont zone of *Eognathodus sulcatus* and the stratigraphic sequence, the Nakaolin Formation are probably of Pragian age [36–40].

## Methods

All the materials were prepared mechanically using a vibro tool with a tungsten-carbide bit or a needle, and some specimens were reversed on latex casts. All specimens were measured with a digital vernier calliper, photographed with a NIKON D3X camera and studied under optical zoom. The phylogenetic character data entry and formatting were performed in Mesquite (version 3.31) [41]. The phylogenetic analysis was conducted using PAUP 4.0a, parsimony analysis package [42].

### Nomenclatural acts

The electronic edition of this article conforms to the requirements of the amended International Code of Zoological Nomenclature, and hence the new names contained herein are available under that Code from the electronic edition of this article. This published work and the nomenclatural acts it contains have been registered in ZooBank, the online registration system for the ICZN. The ZooBank LSIDs (Life Science Identifiers) can be resolved and the associated information viewed through any standard web browser by appending the LSID to the prefix “http://zoobank.org/”. The LSID for this publication is: urn:lsid:zoobank.org:pub: DEE5A348-D73F-454E-B627 -040D3B7ADE1C. The electronic edition of this work was published in a journal with an ISSN, and has been archived and is available from the following digital repositories: PubMed Central, LOCKSS.

## Results

### Systematic paleontology

Subclass Galeaspida Tarlo, 1967

Supraorder Polybranchiaspidida Janvier, 1996

Order Polybranchiaspiformes Liu, 1965

Family Gumuaspidae fam. nov.

urn:lsid:zoobank.org:act:2DBCA7BA-6159-4BE7-A8C4-9659AAC79FC5

**Diagnosis.** Medium to large-sized polybranchiaspiform; head-shield with a robust rostral process like a lute in outline; median dorsal opening ovate or near circular in shape; orbits dorsally placed; pineal opening closed; corner absent; inner corner broad leaf-shaped, end of inner corner beyond the posterior margin of the head-shield; polybranchiaspid-type sensory canal system; posterior supraorbital canal developed and V-shaped; 6–9 pairs of branchial fossae; ornamentation comprising stellate-like or coarse-granular tubercles.

**Type genus.** *Gumuaspis* Wang et Wang, 1992

**Referred genera.** *Platylomaspis* gen. nov., *Nanningaspis* gen.nov., *Pseudolaxaspis* gen. nov.

**Remarks.** A new family Gumuaspidae fam. nov. is erected for polybranchiaspid-like galeaspids including *Gumuaspis*, *Platylomaspis*, *Nanningaspis* and *Pseudolaxaspis*, which bear a rod-like rostral process, but lack laterally projecting corners. The phylogenetic analysis of Galeaspida (see below) shows that the four genera are clustered together to form a monophyletic group, which represents the most primitive clade of Polybranchiaspiformes.

**Fig 2:**
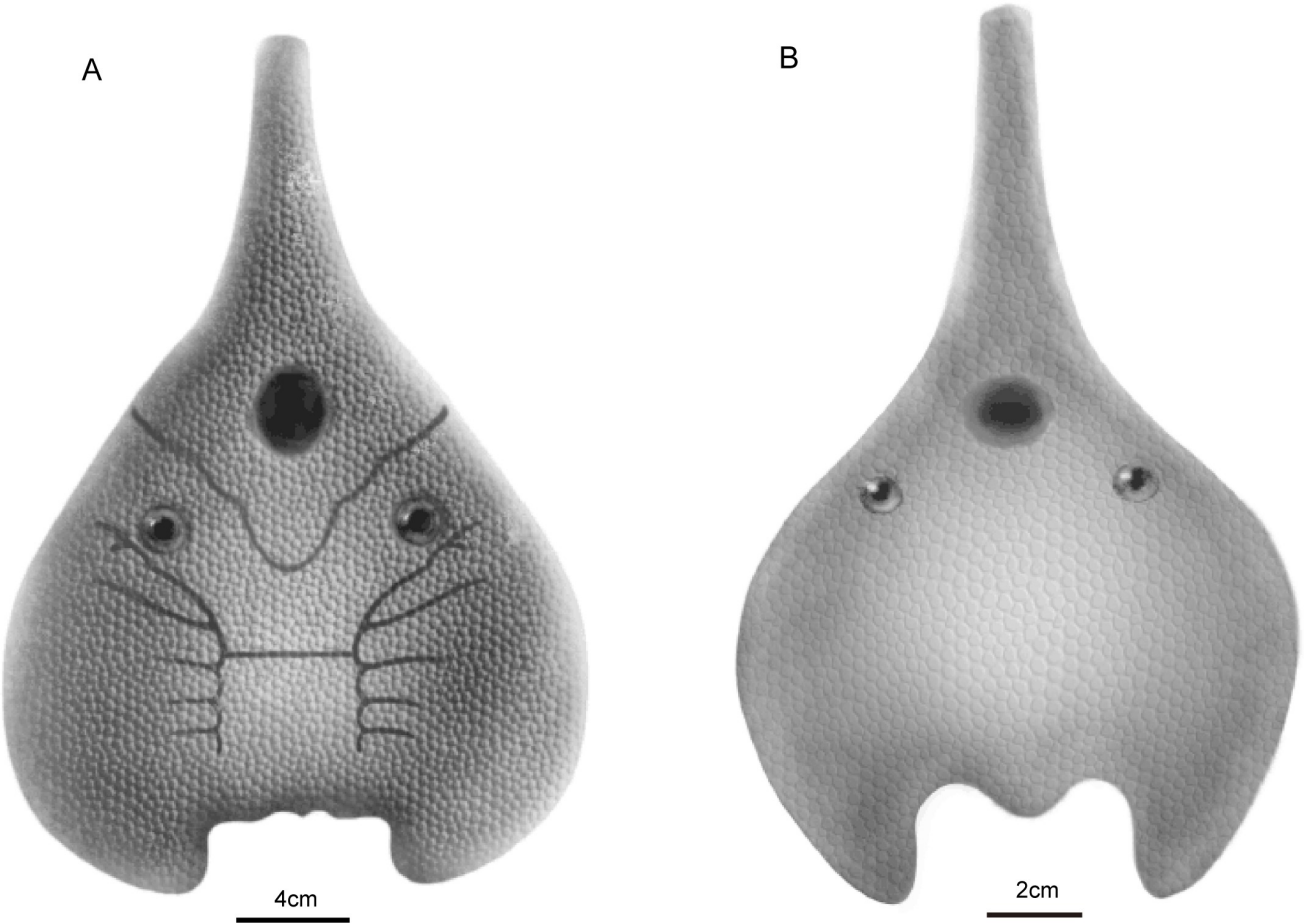
Restoration of *Gumuaspis* (A) and *Pseudolaxaspis* (B)(illustrated by Dinghua Yang).

The type genus *Gumuaspis* was found from the Posongchong Formation (Early Devonian, Pragian) in Wenshan County, Yunnan, China. The new genus *Pseudolaxaspis* gen. nov. is erected for ‘*Laxaspis rostrata*’ from the Xishancun Formation in Qujing, Yunnan. *Pseudolaxaspis* was originally referred to *Laxaspis* as it resembles the type species of *Laxaspis*, *L*. *qujingensis*, in many respects such as the shape and position of the median dorsal opening and orbital opening, the ornamentation of the head-shield, and the end of sensory canals. However, it shows evidently a long rostral process ([43], pl. IV-1), by which ‘*L*. *rostrata*’ is more suggestive of *Gumuaspis rostrata* than *Laxaspis qujingensis* (Fig 2A and 2B) [39]. However, if we assign ‘*L*. *rostrata*’ to *Gumuaspis*, a problem of homonym will be produced since the type species of *Gumuaspis* is named as *G*. *rostrata*. Zhu and Gai suggested an alternative solution to erect a new genus for ‘*L*. *rostrata*’ [4]. Here, we assign a new genus *Pseudolaxaspis* for ‘*L*. *rostrata*’. *Platylomaspis* gen. nov., and *Nanningaspis* gen. nov. are two new forms of Gumuaspidae from the Silurian of the Tarim Basin, Xinjiang, and the Devonian of Nanning, Guangxi respectively.

***Pseudolaxaspis* gen. nov.** (Fig 2B)

urn:lsid:zoobank.org:act:5982E033-4393-4E03-9448-5987597766E1

**Etymology.** *pseud* (Gr.), fake, fraud; *lax* (L.), broad; *aspis* (Gr.), shield, in reference to having the appearance of *Laxaspis*, but not genuine *Laxaspis*.

**Type species.** *Pseudolaxaspis rostrata* (Liu, 1975)

**Diagnosis (emended).** Medium-sized head-shield with a rod-like rostral process; median dorsal opening near circular in outline; orbital opening just behind the level of the posterior margin of the median dorsal opening; end of sensory canal showing a multilateral loop with radial branches; oral fenestra triangular in shape; narrow ventral rim; ornamentation composed of snowflake-like tubercles.

***Pseudolaxaspis rostrata* (Liu, 1975)**(Fig 2B)

*Laxaspis rostrata*: Liu, 1975 [43]

*‘Laxaspis rostrata*’: Liu, 2002 [39]

*‘Laxaspis rostrata*’: Liu et al. 2015 [5]

**Holotype.** An incomplete head-shield, IVPP V4417.

**Type Locality and Horizon.** Liaokuo Park, Qilin District, Qujing City, Yunnan Province, China; Xishancun Formation, Cuifengshan Group, early Lochkovian, Early Devonian.

**Diagnosis.** As for type and only known species.

**Fig 3:**
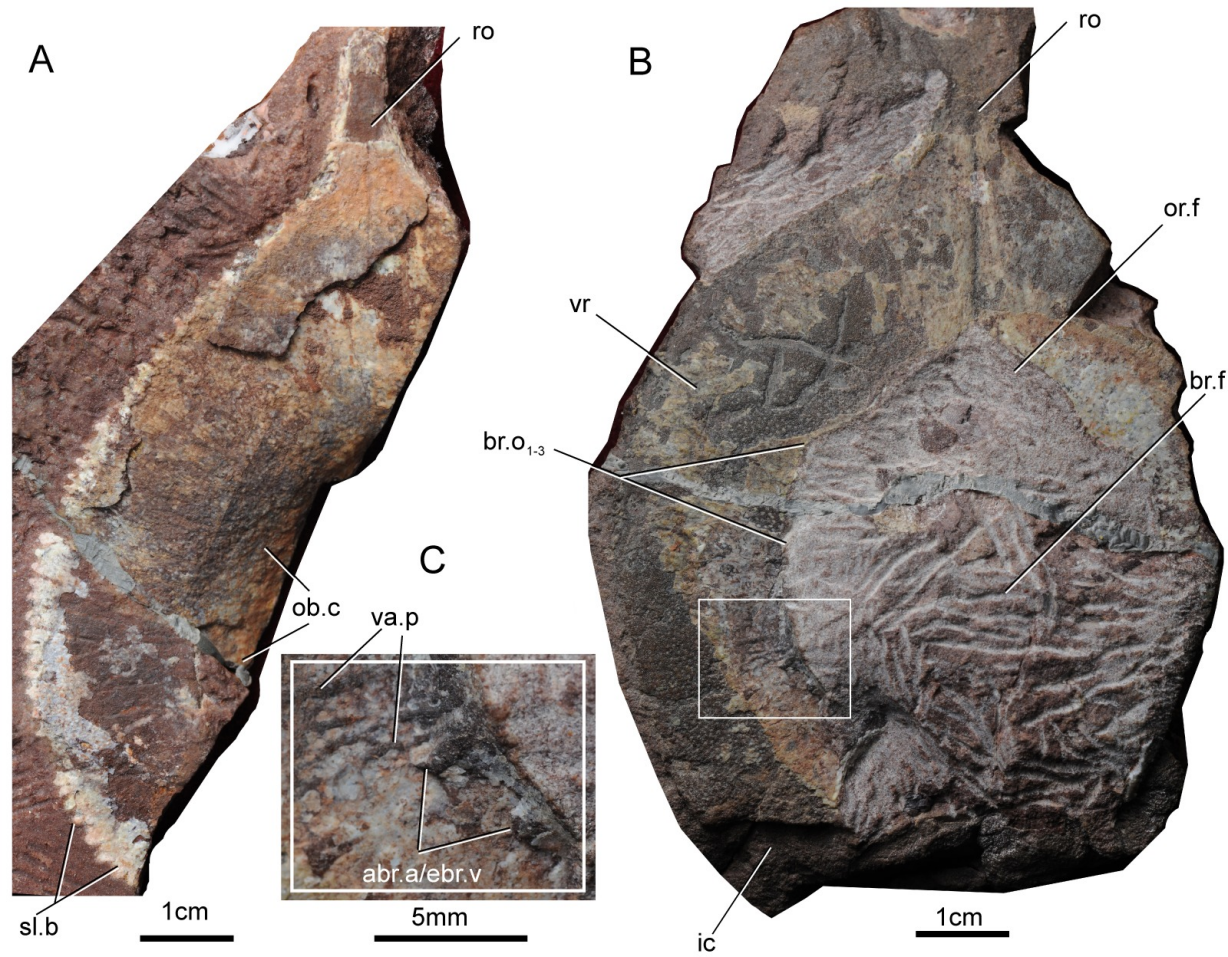
Photograph of *Platylomaspis serratus* gen. et sp. nov. A. an incomplete dorsal head-shield, paratype, GMC V 2415.2; B. a nearly complete ventral head-shield, holotype, GMC V 2415.1; C. close-up of the box region of B, showing the subcutaneous vascular plexi. Abbreviations: abr.a /ebr.v, afferent branchial artery or efferent branchial vein; br.o, branchial opening; br.f, branchial fenestra; ic, inner corner; ob.c, oralobranchial chamber; or.f, oral fenestra; ro, rostral process; sl.b serrated lateral brim; va.p, subcutaneous vascular plexus; vr, ventral rim.

**Fig 4:**
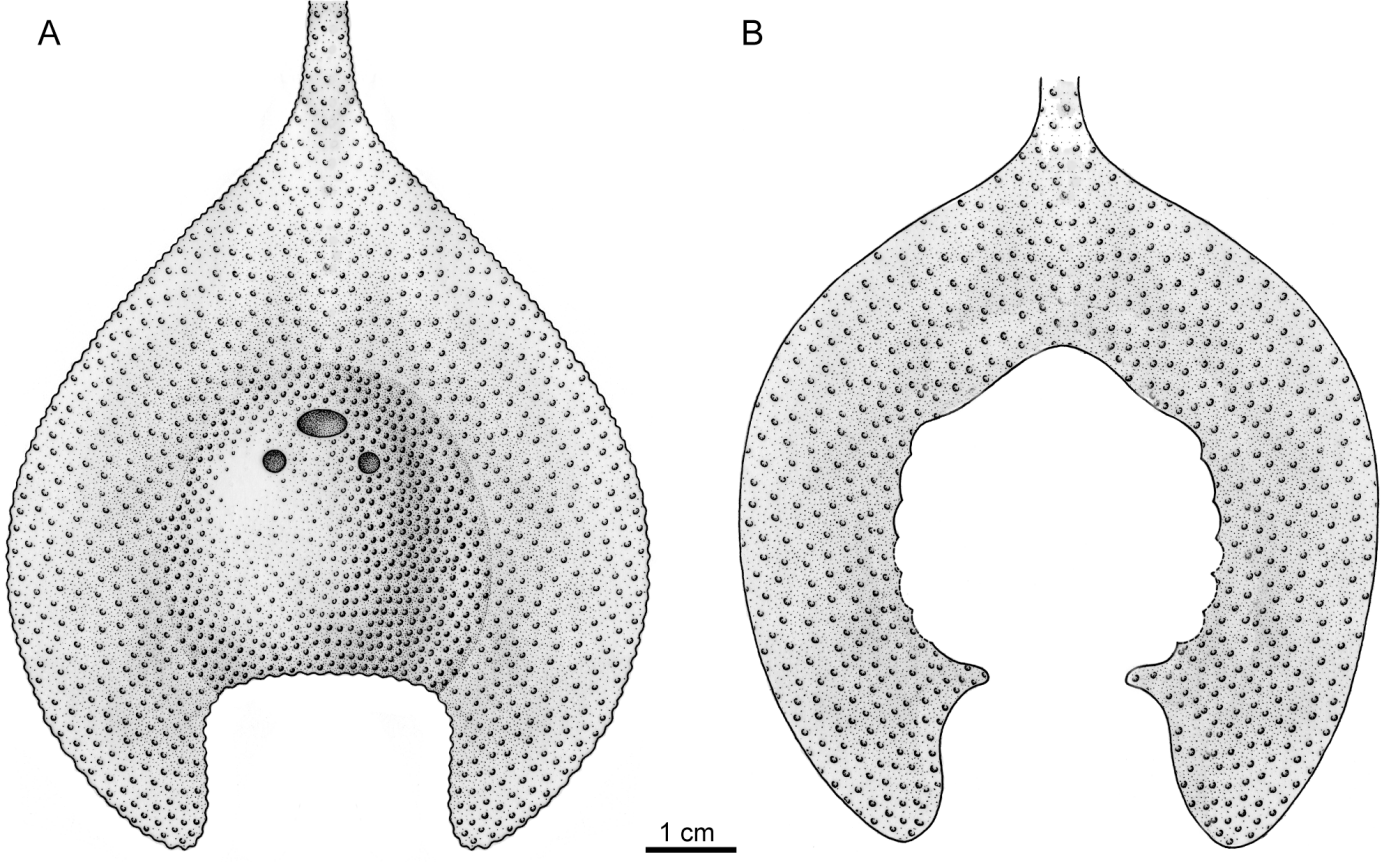
The restoration of *Platylomaspis serratus* gen. et sp. nov. (illustrated by Aijuan Shi). A. in dorsal view; B. in ventral view.

***Platylomaspis* gen. nov.** (Figs 3A–3C, 4A and 4B)

urn:lsid:zoobank.org:act:658494B3-8820-4167-A72F-079C96E39D7D

**Etymology.** *platy* (Gr.), *broad*; lom (Gr.), brim; *aspis* (Gr.), shield, in reference to the broad ventral rim of head-shield.

**Type species.** *Platylomaspis serratus* gen. et sp. nov.

**Diagnosis.** Medium-sized head-shield, 70–80 mm in length (rostral process excluded), and 60–70 mm in width; rostral process rod-like; inner corner broad leaf-shaped; lateral margin of head-shield serrated; ventral rim of head-shield remarkably broad, 18–20 mm in width on each side, approximately one-third the width of the entire head-shield; ventral rim embracing a large pear-shaped oralobranchial fenestra; anterior oral fenestra small, and subtriangular in shape; posterior branchial fenestra large and near circular in shape; ventral rim of either side showing six successive notches for branchial openings; ornamentation composed of coarse-granular tubercles.

***Platylomaspis serratus* gen. et sp. nov.** (Figs 3A–3C, 4A and 4B)

urn:lsid:zoobank.org:act:1A987FA2-2E98-4D10-828D-2F3B20B7FB09

**Etymology.** *serratus* (L.), serrated, in reference to the serrated lateral margin of the head-shield.

**Diagnosis.** As for genus (monotypic).

**Holotype.** A nearly complete ventral head-shield, GMC V 2415.1.

**Paratype.** An incomplete dorsal head-shield, GMC V 2415.2.

**Type locality and horizon.** Tielikewatie village, Kalpin County, Xinjiang Uygur Autonomous Region, China, Tataertag Formation, Telychian, Llandovery, Silurian.

**Fig 5:**
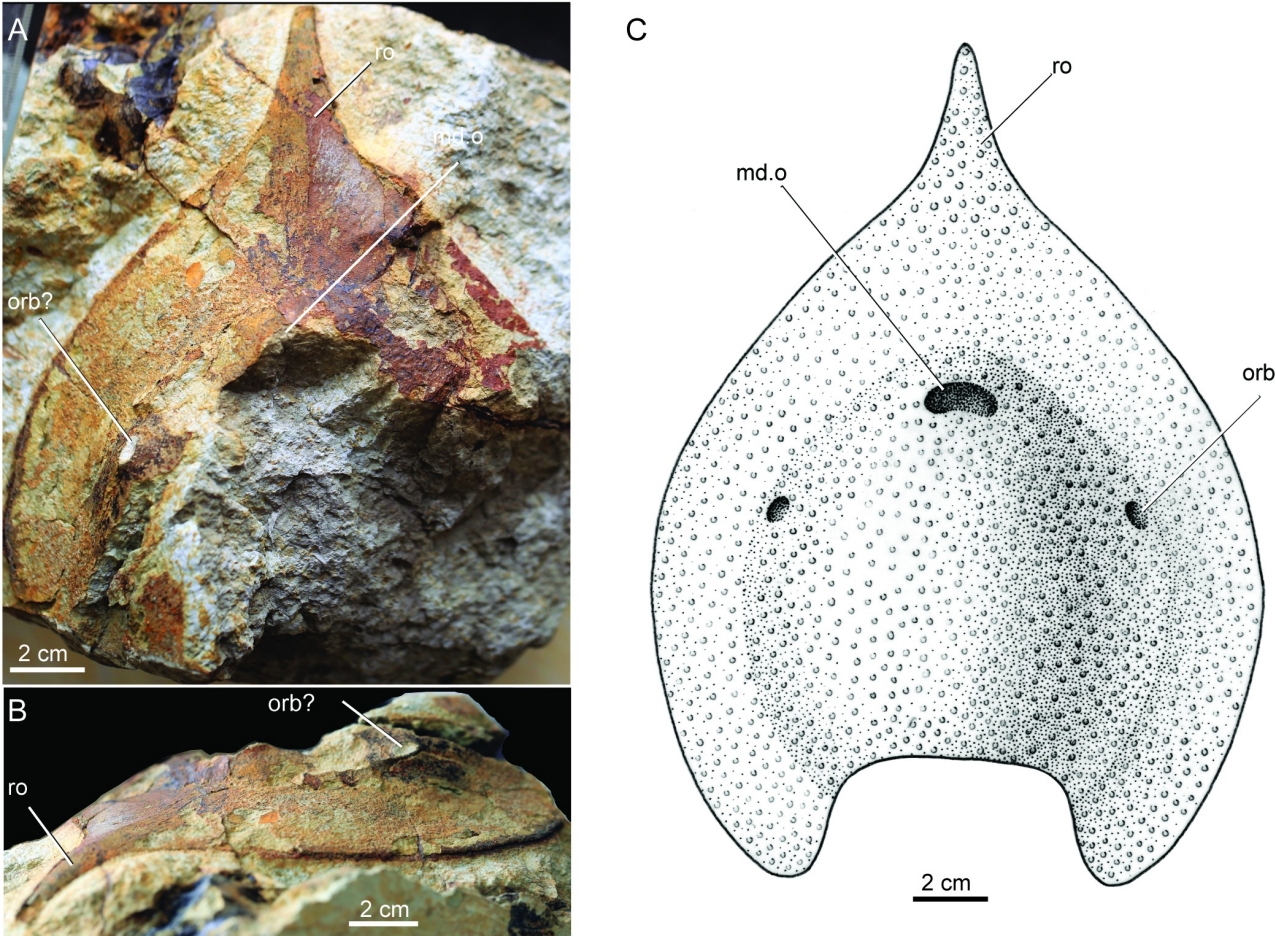
Photograph and restoration of *Nanningaspis zengi* gen. **et sp. nov.** A, B. an incomplete head-shield, holotype, IVPP V24897, A, in dorsal view, B, in anterolateral view; C. restoration (illustrated by Aijuan Shi), in dorsal view. Abbreviations: md.o, median dorsal opening; orb, orbital opening; ro, rostral process.

***Nanningaspis* gen. nov.** (Fig 5A–5C)

urn:lsid:zoobank.org:act:E21ACE38-F115-4ED4-8E4A-486B7B1F4B23

**Etymology.** After the capital city of Guangxi, Nanning, where the fossil was collected, and *aspis* (Gr.), shield.

**Type species.** *Nanningaspis zengi* gen. et sp. nov.

**Diagnosis.** Large-sized head-shield about 225–235 mm in length, and 165–175 mm in width; rostral process rod-like; median dorsal openings and sensory canal system unclear; orbital opening small, and probably located on the dorsolateral margin of head-shield; lateral margin of the head-shield smooth; ventral rim of head-shield remarkably broad, 25–40 mm in width from caudally to rostrally, approximately one-fourth the width of the entire head-shield; ornamentation composed of coarse-granular tubercles.

***Nanningaspis zengi gen*. *et sp*. *nov*.** (Fig 5A–5C)

urn:lsid:zoobank.org:act:E8B34486-AD9B-46B4-BEEA-50BBAA2911C2

**Etymology.** The species is named after Guangcun Zeng, who collected the fossil.

**Diagnosis.** As for genus (monotypic).

**Holotype.** An incomplete head-shield, IVPP V24897.

**Type locality and horizon.** Nanning, Guangxi Zhuang Autonomous Region, South China, Nakaolin Formation, Pragian, Early Devonian.

### Descriptions

#### Platylomaspis serratus

It is a moderate sized polybranchiaspiform. The pear-shaped head-shield tapers rostrally into a long rostral process (ro, Fig 3A and 3B). The length of the head-shield is 70–80 mm (rostral process excluded), the maximum width is about 60–70 mm, and the median length is about 55–60 mm. The maximum width is at the middle part of head-shield, smaller than its length. The rostral process, although incomplete in both specimens, tapers anteriorly as shown by the preserved part. The preserved rostral process is 13.5 mm in the holotype, 16.4 mm in the paratype.

The paratype (Fig 3A) is an incomplete dorsal head-shield with the largest preserved length of 78.3 mm, and the largest preserved width of 45.7 mm. The paratype shows a small dome-shaped depression in the center and a broad flatten marginal band. The roof of the dome-shaped depression occupies the main part of the head-shield, but the median dorsal opening, orbital opening and sensory line system are not preserved on it. The space in the dome-shaped depression encloses the oralobranchial chamber (ob.c, Fig 3A). The broad flat marginal band indicates that *Platylomaspis serratus* has a broad the ventral rim. There are 5–6 serrations every 10 mm along the lateral margin of the head-shield (sl.b, Fig 3A).

The holotype is a nearly complete ventral head-shield (Fig 3B) with the largest preserved length of 65.9 mm, and the largest preserved width of 60.8 mm. The ventral rim on each side is very broad, about 20.0 mm in width, which is approximately one-fourth the width of the entire head-shield. Normally, the ventral rim only accounts for one-tenth of the entire head-shield in most member of Polybranchiaspiformes (e.g. *Polybranchiaspis*, *Pentathyraspis*). Therefore, the exceptional broad ventral rim can be regarded as a diagnostic character of *Platylomaspis*. An incomplete cornual process with the largest preserved length of 8.7 mm is preserved in the left side of the holotype. The cornual process is broad leaf-shaped, and oriented caudally. Zhu (1992) suggested that the true "corner" probably have secondary lost in the Hanyangaspidae and Polybranchiaspidae, and their developed leaf-shaped cornual process was homologous with the inner corner of *Dayongaspis* and eugaleaspids [44]. The shape of head-shield and cornual process of *Platylomaspis* are very similar to that of Polybranchiaspiformes. Therefore, the cornual process of *Platylomaspis* represents the inner corner, and its true corner is absent (ic, Fig 3B).

The ventral rims embrace a large pear-shaped fenestra for the oralo-branchial fenestra, through which the oralo-branchial chamber opens to outside. It remains unknown whether there is a dermal plate covering the oralobranchial fenestra. The anterior oral fenestra (or.f, Fig 3B) looks like an inverted funnel with a maximum width of 20.5 mm, and a maximum length of 15.2 mm. The posterior branchial fenestra (br.f, Fig 3B) is suborbicular in shape with the maximum width of 41.0 mm, and the maximum length of 30.1 mm. There are 5–7 successive notches probably for the external branchial opening (br.o, Fig 3B) along the margin of the branchial fenestra, which exhibits a non-polybranchic condition comparable to that of basal galeaspids *Hanyangaspis*, *Xiushuiaspis*, and the Eugaleaspiformes. In the last two branchial notches, two small canals are preserved probably for the afferent branchial artery or efferent branchial vein (abr.a/ebr.v, Fig 3C) issuing from the marginal vein or artery which is only identified in galeaspids and osteostracans [45]. Posterior to the oralo-branchial fenestra, the ventral rim protrudes inside as a medial process to embrace the fenestra caudally.

The densely-distributed canal network probably for the subcutaneous vascular plexi (va.p, Fig 3C) is discernible between the exo- and endoskeletons on the left part of ventral rim of the holotype. The ornamentation of the head-shield is composed of coarse-granular tubercles as seen in the holotype (Fig 4A and 4B).

#### Nanningaspis zengi

It is a large sized polybranchiaspiform, which is about three times *Platylomaspis serratus* in size. The pear-shaped head-shield tapers rostrally into a long rostral process. The maximum preserved length of the head-shield (including the rostral process) is 183.3 mm, the maximum preserved width is 137.0 mm. The maximum width of the head-shield is estimated to be 167.0 mm. The rostral process is incomplete, but its preserved distal extremity suggests that it tapers anteriorly. The length of the rostral process is 82.8 mm (Fig 5A–5C).

The dorsal side of the head-shield shows a dome-shaped roof with a very broad flatten margin. The median dorsal opening is partly preserved in the anterior part of head-shield, showing vaguely an oval shape as in other polybranchiaspiforms. A small opening in anterolateral part of the dome probably represents the orbital opening. It is elliptic in outline with long axis aligned with the rostro-caudal axis, and the long and short axes are 10.0 mm and 5.3 mm respectively. Compared with other polybranchiaspiforms, the orbital opening of *Nanningaspis* has a more lateral position and faces anterolaterally rather than dorsally (Fig 5A–5C).

Like *Platylomaspis*, *Nanningaspis* has a very broad ventral rim as inferred from the broad flattened margin of the dorsal side of the head-shield. The ventral rim gradually widened from caudal to rostral with the preserved minimum width of 25.8 mm, and the maximum width of 40.6 mm. The lateral margin of the head-shield is smooth without serrations (Fig 5A–5C).

The ornamentation of the head-shield is composed of coarse-granular tubercles (Fig 5A–5C).

### Comparisons

*Platylomaspis* is more comparable to *Gumuaspis* in the pear-shaped head-shield, short rostral process, and leaf-shaped inner corners. *Platylomaspis* differs from *Gumuaspis* mainly by its much broader ventral rim, much larger inner corner, and less branchial fossae. In addition, the lateral margin of the dorsal head-shield is smooth in *Gumuaspis*, but serrated in *Platylomaspis*. The ornamentation of head-shield is composed of stellate tubercles in *Gumuaspis* in contrast to coarse-granular tubercles in *Platylomaspis* (Figs 2A, 3 and 4).

*Nanningaspis* resembles *Gumuaspis* and *Platylomaspis* in bearing a pear-shaped head-shield with a rod-like rostral process and without laterally projecting corner (Figs 2A and 3–5). In comparison to *Gumuaspis*, *Nanningaspis* is more similar to *Platylomaspis* in having a broad ventral rim and coarse-granular tubercles. *Nanningaspis* differs from *Platylomaspis* in having over two times larger sized head-shield (Figs 3–5). Galeaspids display little size variation, e.g. apart from a slight variation in size, no clear growth series has ever been observed from the rich collection of *Polybranchiaspis liaojaoshanensis* in Yunnan and *Shuyu zhejiangensis* in Zhejiang [3, 46]. In addition, the lateral margin of the head-shield is serrated in *Platylomaspis*, but smooth in *Nanningaspis*. Although the median dorsal opening, orbital opening, and sensory canal system remain unclear in both taxa, the available information indicates that the orbital opening of *Nanningaspis* is more laterally set than that of *Platylomaspis*, and probably faces anterolaterally (Figs 3–5).

### Phylogenetic results

To explore the phylogenetic positions of *Platylomaspis* and *Nanningaspis*, we conducted phylogenetic analysis using the dataset of Zhu and Gai [4] with the addition of Character 54, broad ventral rim: absent (0), present (1)(S1 File). The character data entry and formatting were performed in Mesquite (version 3.31). All characters were treated as unordered and weighted equally, as in the earlier versions of this dataset. A basal osteostracan *Ateleaspis* is selected as outgroup for the phylogenetic analysis. The analysis was conducted using PAUP 4.0a, parsimony analysis package, using the heuristic search option (1000 replicates, random addition sequence) [42]. Total number of rearrangements tried = 1.5515e+10. This analysis yielded three equally most-parsimonious trees with a tree length = 134, consistency index (CI) = 0.5038, retention index (RI) = 0.7959 (S2 File).

**Fig 6:**
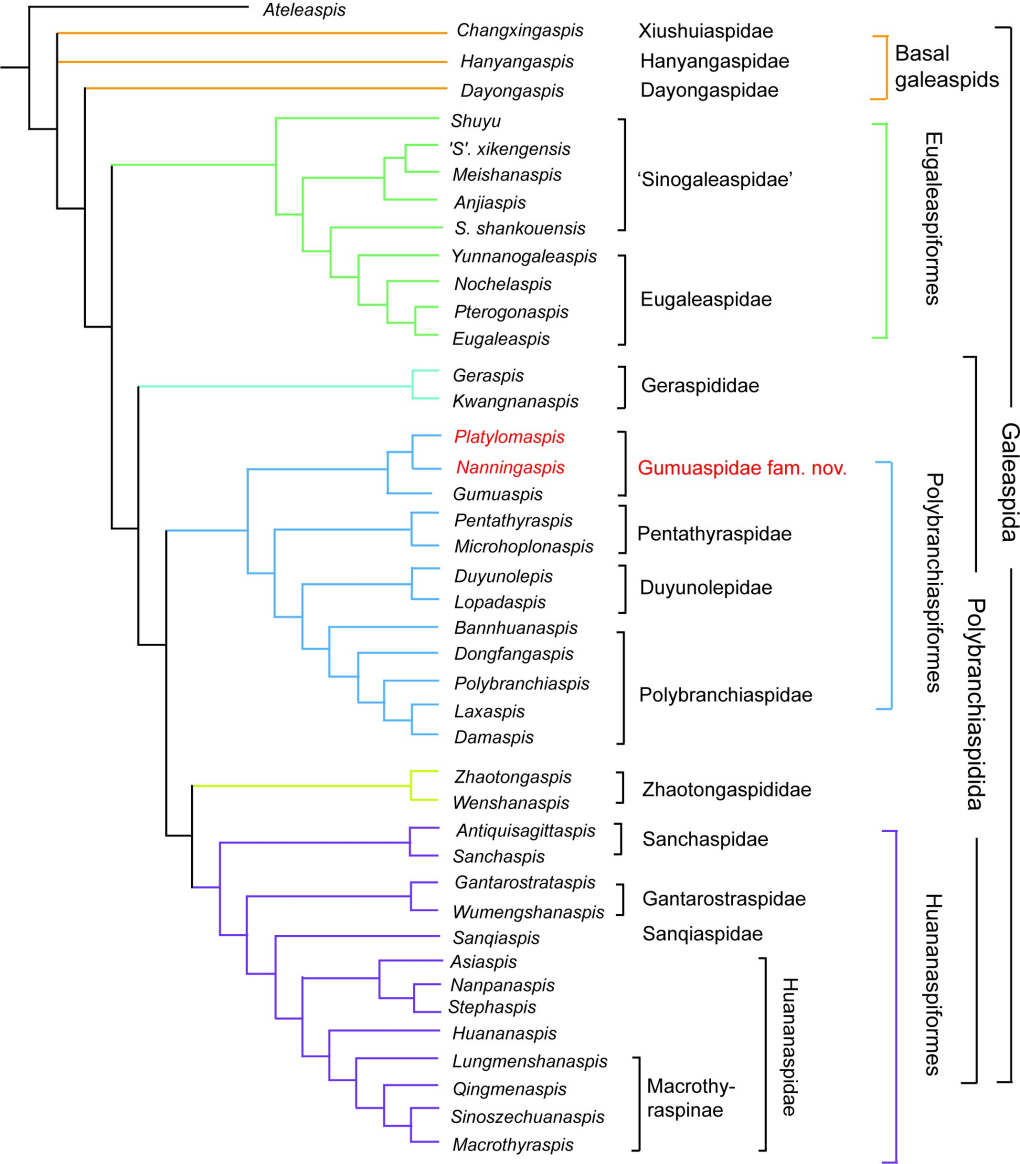
Strict consensus of 3 maximum parsimony trees resulted from the present analysis.

These three equally most-parsimonious trees differ only in the positions of 3 Silurian genera *Hanyangaspis*, *Changxingaspis* and *Dayongaspis* and yield three major clades as defined in earlier works, namely the Eugaleaspiformes, Polybranchiaspiformes and Huananaspiformes. *Platylomaspis* and *Nanningaspis* fall into the Polybranchiaspiformes. Within Polybranchiaspiformes, *Platylomaspis* is resolved as the sister group of *Nanningaspis* by the synapomorphy of the broad ventral rim. They are consistently clustered with *Gumuaspis* to form a monophyletic group, Gumuaspidae fam. nov., and positioned at the base of Polybranchiaspiformes (Fig 6).

## Discussion

### Temporal and palaeobiogeographic distribution of Polybranchiaspiformes

**Fig 7:**
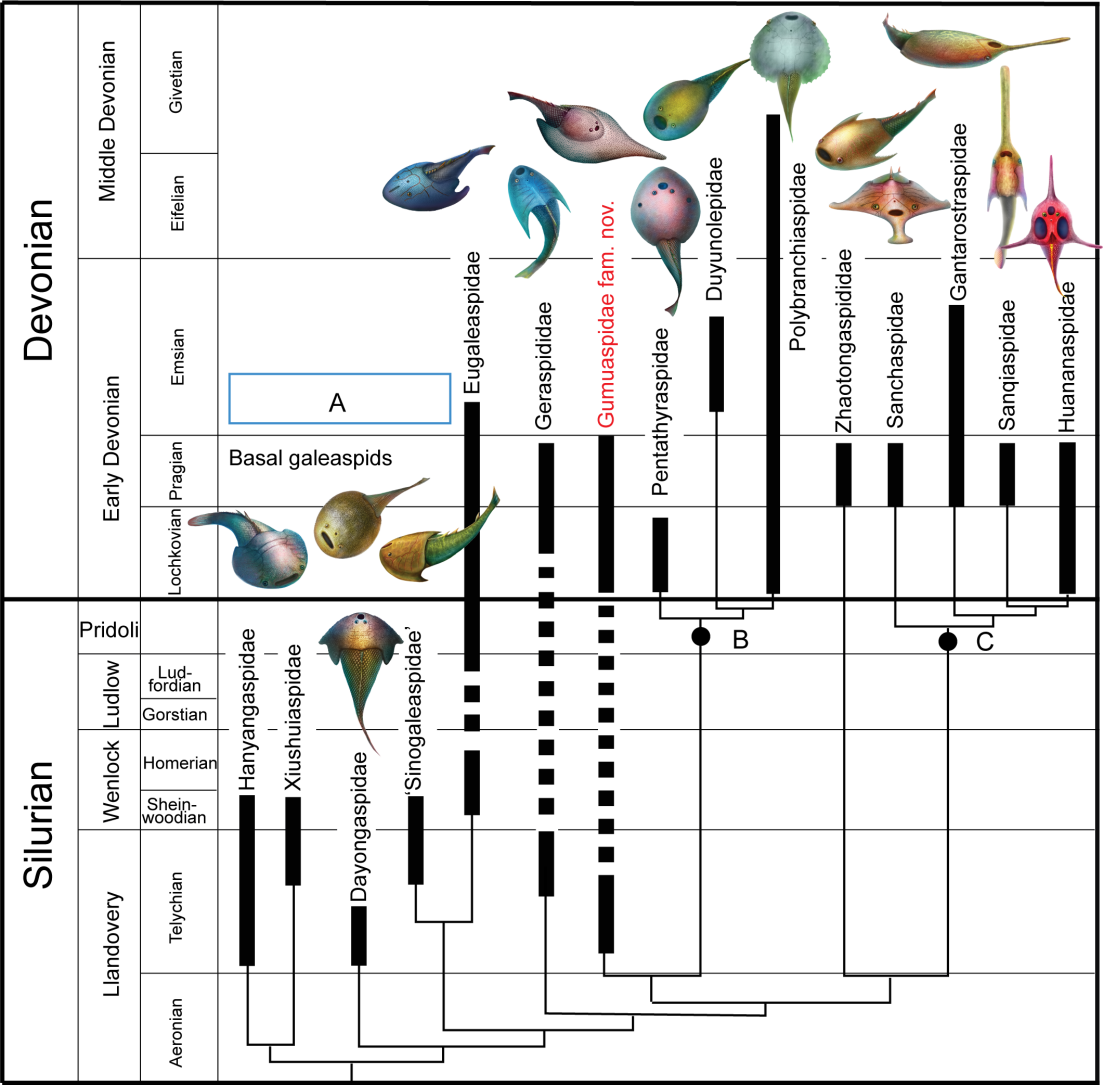
A phylogenetic tree of galeaspid families projected against stratigraphy. (Solid columns represent known time ranges, thin lines represent ‘ghost lineages’).

Our phylogenetic analysis indicates that the Gumuaspidae occupies the most basal position of Polybranchiaspiformes. The Polybranchiaspiformes is a highly diverse group of galeaspids, which was assumed to occur from the Lochkovian to the Eifelian [4, 47]. The earliest fossil record of Polybrachiaspifomes was from the Xishancun Formation (Lochkovian) including *Pentathraspis*, *Polybranchiaspis*, *Laxaspis*, *Damaspis*, *Pseudolaxaspis*, *Diandongaspis*, and *Altigibbaspis* [43, 48–51]. The high diversity of Polybrachiaspifomes in the Xishancun Formation indicated that the Polybrachiaspifomes should have differentiated earlier. The finding of *Platylomaspis* from the Telychian of Llandovery, Silurian (~438 million years ago) extends the earliest occurrence of Polybranchiaspiformes backward about 19 million years (Fig 7). By the Pragian, the diversity of Polybranchiaspiformes suddenly declined. Except for the Gumuaspidae, there are only two unusually large-sized polybranchiaspiforms: *Bannhuanaspis vukhuci* from the uppermost part of the late Lochkovian to the early Pragian (Si Ka Formation of Bac Bo, Vietnam) and *Dongfangaspis major* from the Pragian (Pingyipu Formation of Sichuan) survived [43, 52]. The Gumuaspidae, as the longest lasting galeaspid family from the Telychian to the Pragian (~407 million years ago), witnessed the three major radiations of galeaspids: i.e. basal galeaspids plus Eugaleaspiformes (box A, Fig 7) in the Telychian, Polybranchiaspiformes in the Lochkovian (Node, B, Fig 7) and Huananaspiformes in the Pragian (Node, C, Fig 7).

The findings of *Platylomaspis* and *Nanningaspis* also have important palaeobiogeographic significance. For the Galeaspida, a correlation between phylogeny and palaeobiogeography is observed, e.g. a close association between Tarim and the southeastern China region is reconstructed by the phylogeny of ‘basal’ galeaspids [53]. Over the last decades, the polybranchiaspiforms have been assumed to be endemic to southern China and northern Vietnam, never found in Tarim [4, 47]. The earliest occurrence of *Platylomaspis* in the Silurian Tarim Basin expands the distribution of polybranchiaspiforms from South China to Tarim (Fig 1A). As galeaspids are heavily armoured bottom-dwellers with limited locomotory and dispersal capabilities, the continents and open oceans became the major obstacles for their migration and dispersal [53, 54]. Geological and paleontological evidence indicates that the South China, the North China and the Tarim blocks were very close during the Llandovery of Silurian. They were neighboured each other, and shared a highly endemic vertebrate fauna, namely “the Pan-Cathaysian Galeaspid Fauna" [54]. The finding of *Platylomaspis* in Tarim and *Nanningaspis* in Guangxi corroborates that a united Tarim-South China Block existed in the Llandovery and Wenlock of Silurian [18, 20, 54], and provides a potential route for early polybranchiaspiforms migrating from the Tarim Block to the South China Block. The united block was probably divided into two blocks in the Ludlow or later, when the Tarim Block began to drift away to the northwest [18].

### Ecomorphological implications

*Platylomaspis* and *Nanningaspis* are characterized by a pear-shaped head-shield with a rostral process and a unique broad ham-brim-like ventral rim. The rostral process, which was regarded as a diagnostic character of Huananaspiformes [55] are also present in other two major groups of galeaspids, e.g. Gumuaspidae in Polybranchiaspiformes, Eugaleaspidae in Eugaleaspiformes [44, 56]. The rostrum of galeaspids might have the same functions as the rostrum of other agnathans that lived under similar environmental conditions, e.g. boreaspidid osteostracans, pituriaspids, and amphiaspidid and pteraspidid heterostracans [3, 57–59]. It may have a sensory function in search of food or a hydrodynamic function [60, 61]. However, the hypothesis of the protective function of the rostrum by scaring predators in osteotracans proposed by Janvier [62] are probably not applicable to Gumuaspidae as it lacks other processes, e.g., the laterally directed cornual processes which can largely intensify the effect of protective function. The recurrent evolution of similar rostral processes in various agnathan groups probably indicates similar ecological pressures upon the clades and diversifications into comparable niches [63].

**Fig 8:**
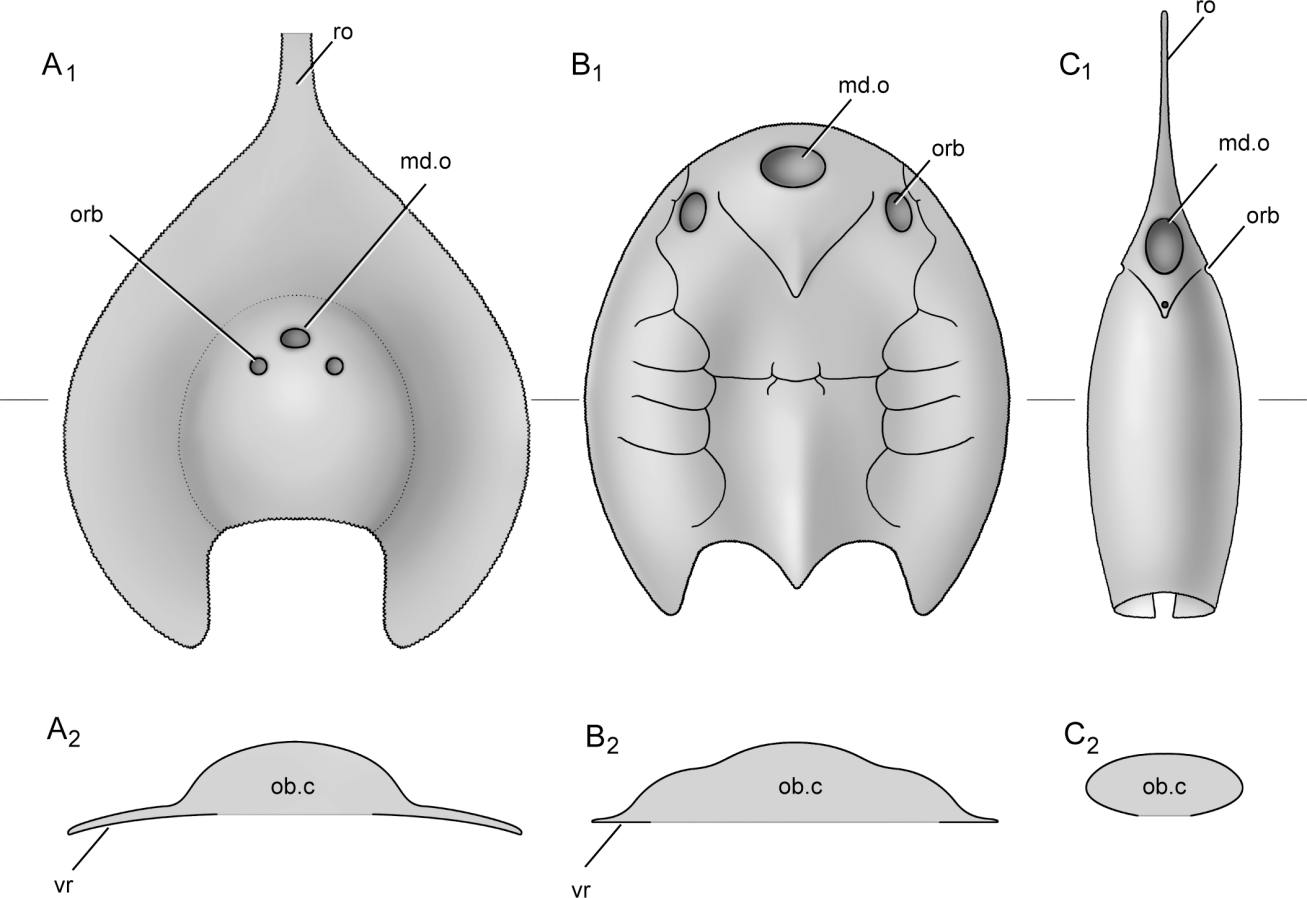
The modification of the ventral rim in galeaspids (illustrated by Dinghua Yang). A. a very broad ventral rim in *Platylomaspis*; B. a normal ventral rim in *Polybranchiaspis*; C. ventral rim absent in *Rhegmaspis*. A_1_–C_1_, in dorsal view; A_2_–C_2_, a cross section through the head-shield at the level indicated by the line in A_1_–C_1_. Abbreviations: md.o, median dorsal opening; ob.c, oralobranchial chamber; orb, orbital opening; ro, rostral process; vr, ventral rim.

**Fig 9:**
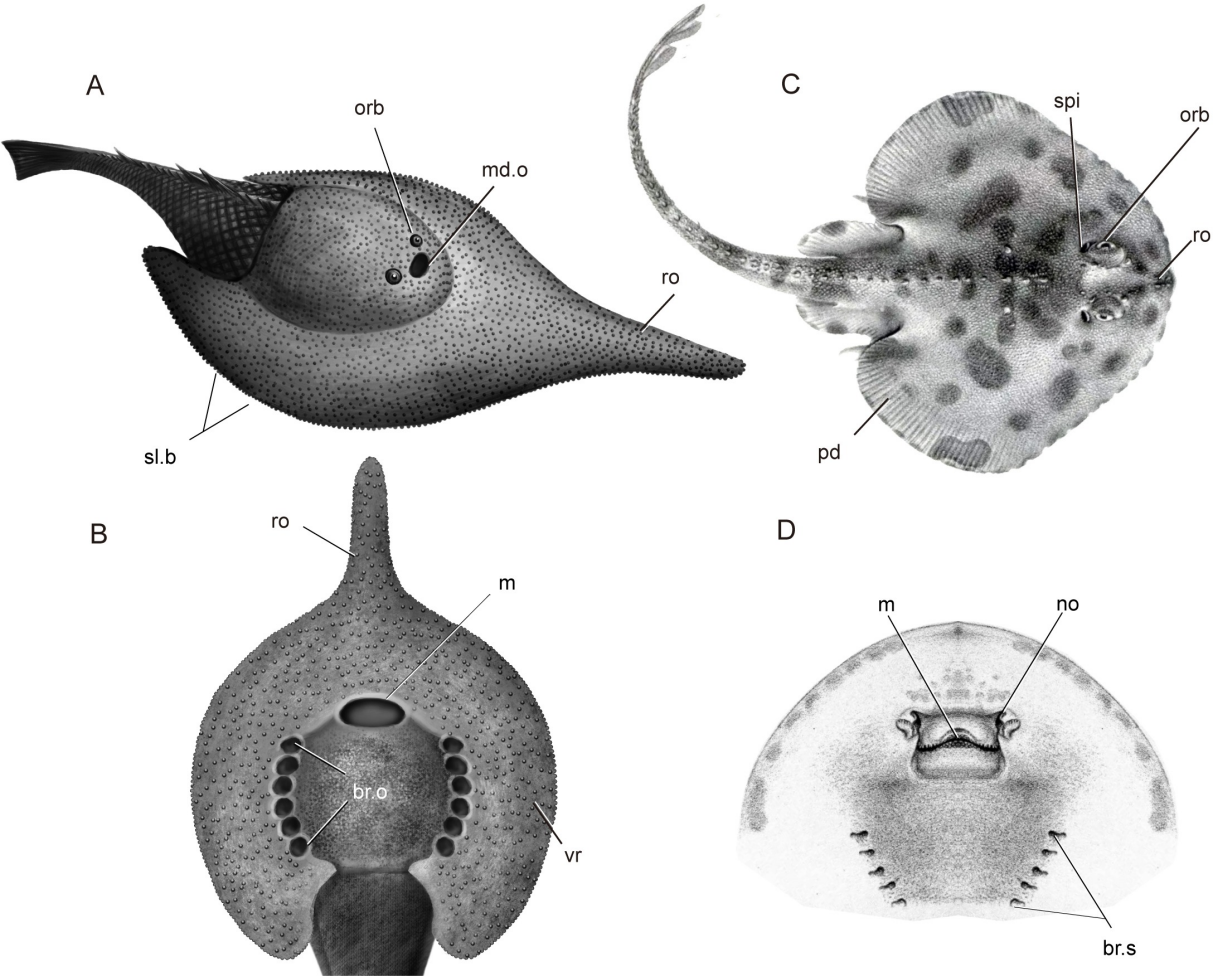
The morphological convergence between galeaspids and morden rays probably for the half burrowing habit. A, B. restoration of galeaspid *Platylomaspis* (illustrated by Dinghua Yang), A, in antero-lateral view, B ventral view; C, D. a modern ray *Rajella fyllae* (modified after Lütken [65]), C, in dorsal view, D, in ventral view. Abbreviations: br.o, branchial opening; br.s, branchial slist; m, mouth; md.o, median dorsal opening; no, nostril; orb, orbital opening; pd, pectoral disc; ro, rostral process; sl.b, serrated lateral brim; spi, spiracle; vr, ventral rim.

The wide-brimmed head-shield is a diagnostic character uniquely shared by *Platylomaspis* and *Nanningaspis* (Fig 8A). The morphological adaptations of galeaspids, e.g. a strongly flattened head-shield, dorsally-positioned eyes and 'nostril', suggested that they are coastal bottom-dwellers living in the habitats close to palaeocontinental margin, including delta and estuary [3, 54]. Like modern Cottidae and Gobiesocidae, the strongly armored and flattened head-shield of galeaspids provides greater density and helps the fish maintain contact with the substratum in moderate currents without a significant energetic cost [9, 64]. Therefore, the extreme flattening of the head-shields strengthened by the wide ventral rim in *Platylomaspis* and *Nanningaspis* at least can help them better adapt to the benthic habitat or have a selective advantage when living in an environment dominated by strong currents. Here, we further show that the wide-brimmed head-shield with a rod-like rostral process in *Platylomaspis* and *Nanningaspis* is probably analogous to the pectoral disc and long duck-billed snout in modern rays (Fig 9), e.g. *Dasyatis* and *Rajella* [65]. The head shape of the latter facilitates them to burrow underneath the sands in order to avoid predators and wait for feeding opportunities.

**Fig 10:**
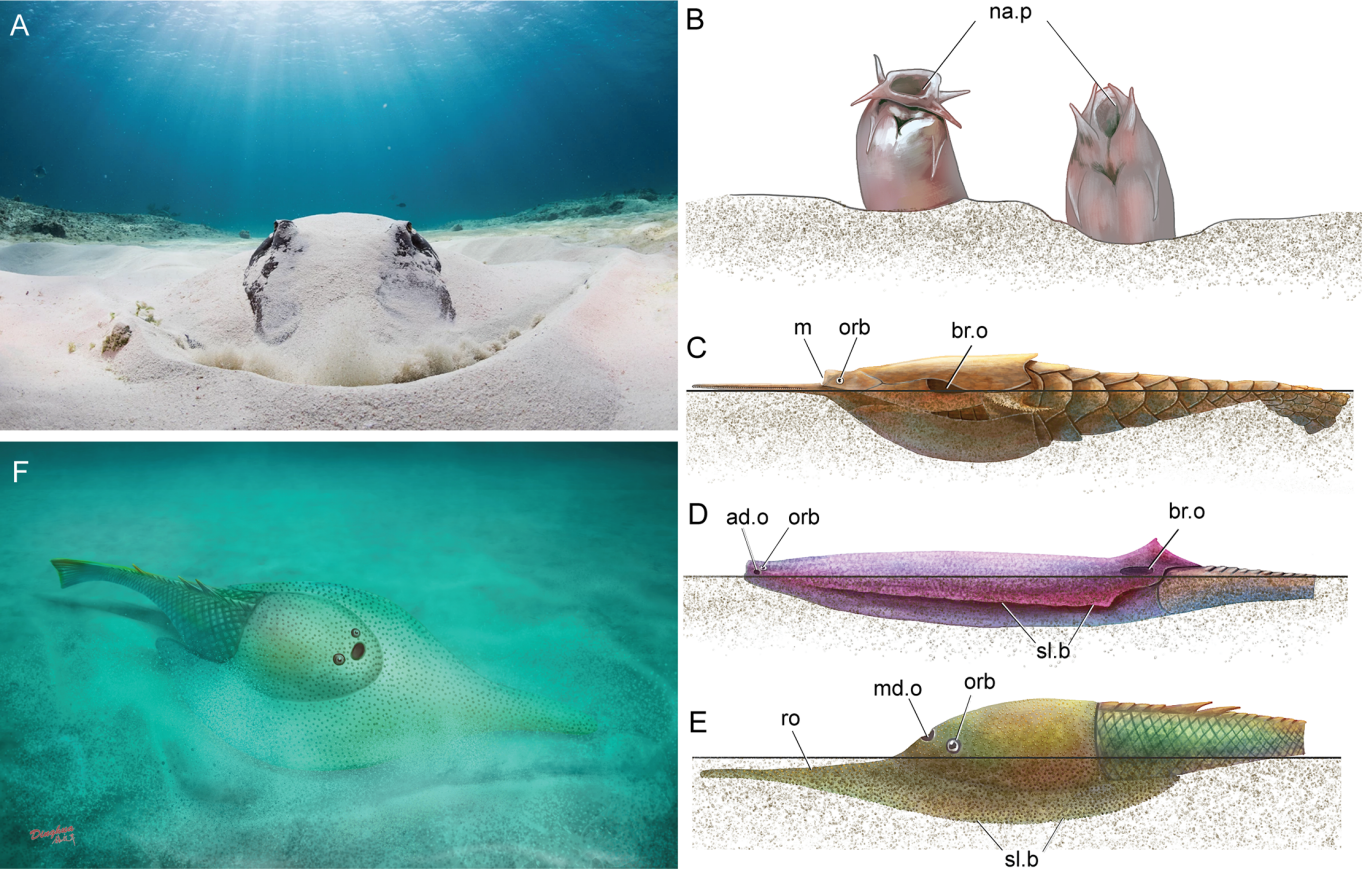
Adaptions of the water intake device in the half burrowing fishes. A. a modern southern stingray *Dasyatis* using its spiracle for the intake of respiratory water when buried in sand (photo courtesy by Eugene Kitsios/Bimini Biological Field Station Foundation, http://www.kitsios-photography.com); B. the modern Pacific hagfish *Eptatretus* using its nasopharyngeal duct for the intake of respiratory water when buried in mud; C. the pteraspid heterostracan *Doryaspis* probably using its dorsal set mouth for the intake of respiratory water when buried in sand; D. the amphiaspid heterostracan *Kureykaspis* using its adorbital opening for the intake of respiratory water when buried in sand; E, F. the gumuaspid galeaspid *Platylomaspis* using its median dorsal opening (nostril) for the intake of respiratory water when buried in sand (B–F illustrated Dinghua Yang). Abbreviations: ad.o, adorbital opening; br.o, common external branchial opening; m, mouth; md.o, median dorsal opening; na.p, nasopharyngeal duct; orb, orbital opening; ro, rostral process; sl.b, serrated lateral brim; spi, spiracle.

Galeaspids have a remarkable character combination to make breathing and feeding more effective. Like modern rays, galeaspids have their mouth and gill openings on the ventral side of head-shield (Fig 9B, m, br.o), but have a diagnostic large median dorsal opening for the nostril located on the anterodorsal side of head-shield (md.o, Fig 9A). The synchrotron radiation X-ray tomographic study revealed that the large median dorsal opening penetrated the roof of oral cavity to communicate with the oral cavity and served for the main water intake device [1]. The diagnostic median dorsal opening of galeaspids is probably analogous to the spiracle in modern rays (Fig 9C, spi) through which the oxygenated water is inhaled. After oxygen is exchanged in gills, the deoxygenated water is exhaled though the ventral branchial opening. This is a useful adaptation for a benthic animal because it can respire oxygen without any need to move and have its mouth free for feeding. Janvier [3] and Pernègre [66] considered that the water intake device in some jawless fishes, e.g. the nasopharyngeal duct in hagfishes (na.p, Fig 10B), the dorsally positioned mouth (m, Fig 10C) and the adorbital openings (ad.o, Fig 10D) in pteraspid and amphiaspid heterostracans, as well as the median dorsal opening in galeaspids (md.o, Fig 10E) probably linked with the partial burrowing habits.

However, this hypothesis of the partial burrowing habit is not suitable for most galeaspids because their median dorsal openings are so subterminal, or even terminal, that the silt and mud would pour into oralobranchial chamber when they were buried to inhale water (Fig 8B). Being choked while hiding in sands means that the galeaspids cannot hide themselves in sands and are subject to the danger of predators. By contrast, the wide-brimmed head-shield and rostral process in *Platylomaspis* and *Nanningaspis* had set the median dorsal opening and orbital openings highly in the middle of the head-shield, which not only makes the head-shield easy to be buried in silt or mud, but also can keep eyes and the large median dorsal opening outside for vision and respiration (Fig 10E and 10F). In this case, the rostral process could possibly function as a detector of potential food in silt or mud like the long duck-billed snout of modern rays. In addition, the coarse ornamentation and serrated lateral margin in *Platylomaspis* would play a role in resisting the tendency to slide backwards while moving forward through the sediment as proposed in some ctenaspid heterostracans (sl.b, Fig 10D and 10E) [67, 68]. Hence, we propose that the unique wide-brimmed head-shield together with the rostral process in *Platylomaspis* and *Nanningaspis* is most likely to be a special adaptation for a semi-infaunal benthos, representing a partial burrowing habit in galaeaspids. With the semi-infaunal habit, *Platylomaspis* and *Nanningaspis* were better hidden from predators than epifaunal galeaspids and could stay in moderate currents with less energetic cost.

**Fig 11:**
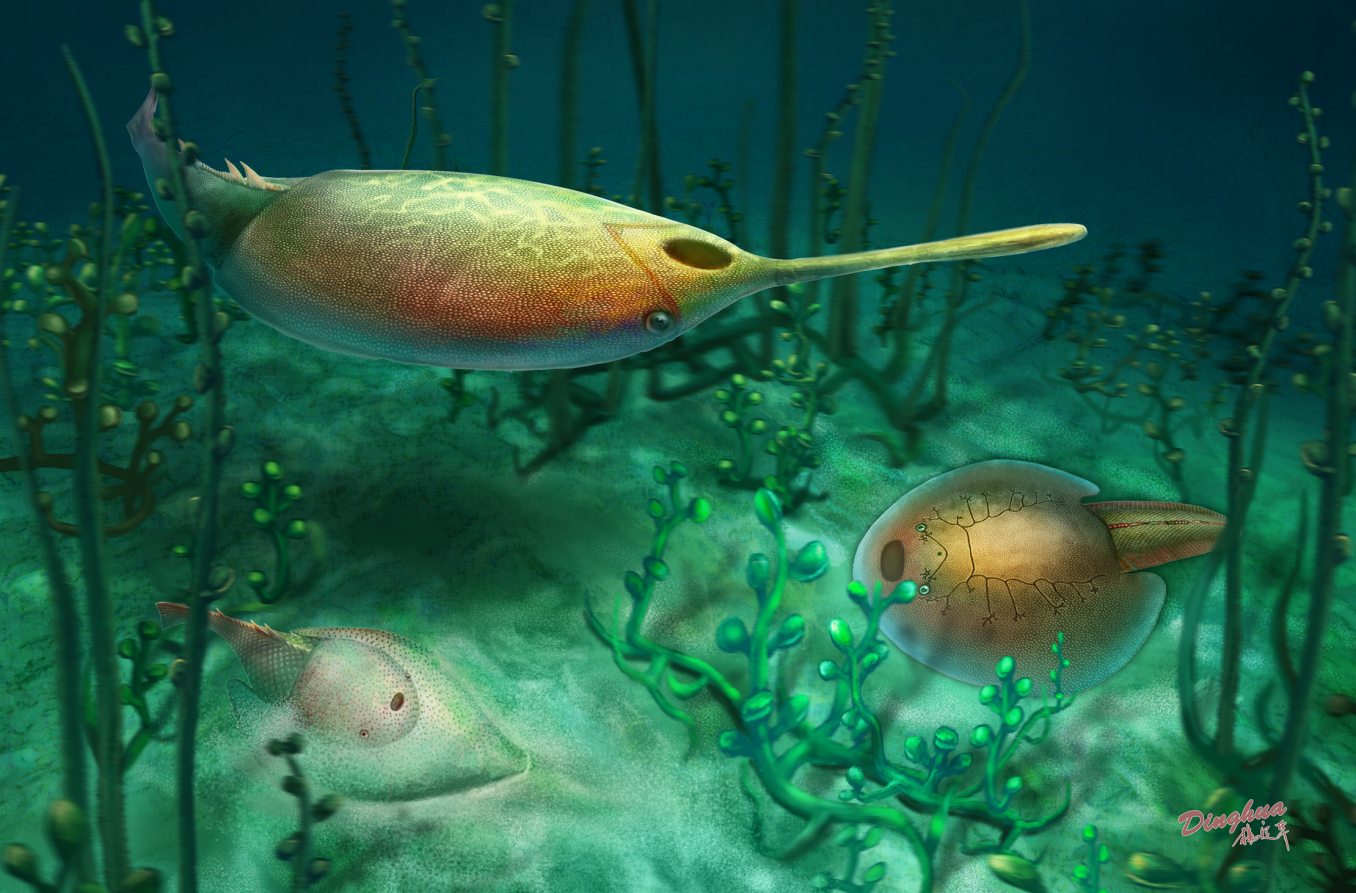
Ecological restoration of galeapids during the Pragian showing three different types of habits (illustrated by Dinghua Yang). Bottom: *Nanningaspis* lived in a semi-infaunal benthic (half buried) habit; middle: *Dongfangaspis* lived in a epibenthic habit; up: *Rhegmaspis* lived in a suprabenthic (nektonic) habit.

Recent studies indicated that the ventral rim of galeaspids had undergone an opposite modification in the Gantarostrataspidae [9], a clade of Huananaspiformes. Compared to the very broad ventral rim in *Platylomaspis* and *Nanningaspis*, the ventral rim of the Gantarostrataspidae is largely reduced, and even completely lost to form a streamlined torpedo-like head-shield (Fig 8C) to minimize the water drag by reducing the magnitude of the pressure gradient over the body [9]. This indicates that some galeaspids such as *Rhegmaspis* probably became more effective swimmer for a suprabenthic or nektonic habit (Fig 11, up). As such, the long rostral process was probably used as a scraper to plow the bottom in a relatively high speed to stir digestible materials for a more efficient filtering than the epibenthic galeaspids [9]. The discoveries of *Nanningaspis* from the Nakaolin Formation, *Dongfangaspis* from the Pingyipu Formation, and *Rhegmaspis* from the Posongchong Formation indicate that the demersal galeaspids had further developed three different kinds of habit types: semi-infaunal benthic (half buried) (Fig 10 bottom), epibenthic (Fig 11, middle), and suprabenthic (nektonic) (Fig 11, up) habits to occupy different vertical ecological niches by the Pragian of the Early Devonian. Coupled with such a habit differentiation, galeaspids reached the peak of their diversity during the Pragian[69].

## Supporting information

S1 File The nexus file of the data matrix with 41 ingroup taxa and 54 characters. (LOG)

S2 File The log file of the phylogenetic analysis. (NEX)
